# Protective Mechanisms of the Mitochondrial-Derived Peptide Humanin in Oxidative and Endoplasmic Reticulum Stress in RPE Cells

**DOI:** 10.1155/2017/1675230

**Published:** 2017-07-26

**Authors:** Leonid Minasyan, Parameswaran G. Sreekumar, David R. Hinton, Ram Kannan

**Affiliations:** ^1^Department of Pathology, Keck School of Medicine of the University of Southern California, Los Angeles, CA, USA; ^2^Arnold and Mabel Beckman Macular Research Center, Doheny Eye Institute, Los Angeles, CA, USA; ^3^Department Ophthalmology, USC Roski Eye Institute, Keck School of Medicine of the University of Southern California, Los Angeles, CA, USA

## Abstract

Age-related macular degeneration (AMD) is the leading cause of severe and irreversible vision loss and is characterized by progressive degeneration of the retina resulting in loss of central vision. The retinal pigment epithelium (RPE) is a critical site of pathology of AMD. Mitochondria and the endoplasmic reticulum which lie in close anatomic proximity to each other are targets of oxidative stress and endoplasmic reticulum (ER) stress, respectively, and contribute to the progression of AMD. The two organelles exhibit close interactive function via various signaling mechanisms. Evidence for ER-mitochondrial crosstalk in RPE under ER stress and signaling pathways of apoptotic cell death is presented. The role of humanin (HN), a prominent member of a newly discovered family of mitochondrial-derived peptides (MDPs) expressed from an open reading frame of mitochondrial 16S rRNA, in modulation of ER and oxidative stress in RPE is discussed. HN protected RPE cells from oxidative and ER stress-induced cell death by upregulation of mitochondrial GSH, inhibition of ROS generation, and caspase 3 and 4 activation. The underlying mechanisms of ER-mitochondrial crosstalk and modulation by exogenous HN are discussed. The therapeutic use of HN and related MDPs could potentially prove to be a valuable approach for treatment of AMD.

## 1. Age-Related Macular Degeneration

Age-related macular degeneration (AMD) is a progressive degenerative retinal disease that impairs visual acuity and causes irreversible central vision loss. In the developed world, AMD is the leading cause of blindness of the geriatric population [1]. In the United States alone, approximately 11 million people suffer from AMD, and this number is expected to double to 22 million by the year 2050 [2]. To date, no viable treatments to cure AMD exist and the need for novel therapeutics is becoming increasingly vital to circumvent the economic burden of inflating health care costs resulting from an increasing patient population.

Clinically, AMD is classified into two phases, an early asymptomatic phase containing drusen deposits and areas of hyper- or hypopigmentation and an advanced/late phase marked by profound vision loss and retinal degeneration [3]. The early phase of the disease may progress into either of the two advanced AMD subtypes: geographic/atrophic AMD (“dry AMD” or GA) with loss of retinal pigment epithelium (RPE) cells and photoreceptors or exudative/neovascular AMD (or “wet AMD”), which is distinguished from the former by the presence of choroidal neovascularization (CNV) [4]. These subtypes are not mutually exclusive as patients may exhibit characteristics of both dry and wet AMD in one eye, dry in one eye and wet in the other, and even the evolution of dry to wet AMD and vice versa [5].

Though the etiology of AMD is enigmatic due to its multifactorial nature, the dynamic interplay of several pathological processes has been well documented in the macula and is associated with a plethora of risk factors including aging, genetic, metabolic, and environmental such as smoking and phototoxicity [4, 6]. Genome-wide association studies (GWAS) comparing AMD patients and controls identified 52 independently associated common and rare variants distributed across 34 loci [7].

The most notable pathogenic processes in AMD include (1) the accumulation of discrete drusen deposits (composed of partially degraded oxidized products) between the RPE and Bruch membrane (BM), (2) intracellular accumulation of lipofuscin granules in the RPE and its constituent bis-retinoid N-retinylidene-N-retinylethanolamine (A2E), (3) chronic local inflammation, (4) innate immune activation of the complement system by the inflammasome nod-like receptor-P3 (NLRP3), (5) mtDNA damage, and (6) oxidative and endoplasmic reticulum stress. In addition, neovascular AMD is characterized by abnormal growth of immature and leaky choroidal vessels through BM which is associated with increased expression of vascular endothelial growth factor (VEGF) [1].

## 2. Critical Role of RPE in Retinal Function

The RPE cells are a postmitotic polarized cuboidal monolayer separating the photoreceptors of the neural retina and the vascular choroid. They are an essential component of the outer blood-retinal barrier and are critical for maintaining the eye as a site of relative immune privilege. Additional functions include mediating bidirectional fluid and ion transport, phagocytosis of shed photoreceptor outer segments (POS), synthesizing and regulating the subretinal extracellular matrix (ECM), secreting growth factors, and recycling rhodopsin as part of the visual cycle [8].

Prevailing knowledge implicates the exacerbation of oxidative stress (OS) coupled with the attenuation of adaptive defense responses with aging in the pathogenesis of macular lesions, and more recently, the crosstalk between oxidative stress (OS) and endoplasmic reticulum (ER) stress has attracted much attention [9–13].

Irrespective of what the primary pathogenic insult may be, RPE dysfunction is a hallmark of AMD with a growing body of evidence suggesting that mitochondrial dysfunction, particularly mitochondrial DNA (mtDNA) damage resulting from aging and the OS-ER stress interface, may be critical [1, 14]. A recent report by Terluk et al. [15] showed that mtDNA damage is limited to the RPE, and similar mtDNA damage is found in the macular and peripheral RPE. Further, the study revealed that mtDNA damage is limited to discrete regions of the mitochondrial genome, including the control region containing *cis*-elements responsible for mitochondrial transcription and replication [15]. Other damaged regions of the mitochondrial genome include genes for the 16S and 12S ribosomal RNAs and 8 of 22 tRNAs. The 16S rRNA region also produces mt-derived peptides (MDPs), including humanin, and small humanin-like peptides (SHLPs) [15, 16].

This review will discuss the emerging concept of MDPs and their homeostatic functions in RPE cells. It will predominantly focus on the MDP humanin (HN) and its modulation of retinal OS and ER stress, with emphasis to ER-mitochondrial crosstalk in RPE. Information on these MDPs may provide insight into developing novel AMD therapeutics in conjunction with other agents for combination-based therapies.

## 3. Evolution and Function of Mitochondria

Mitochondria are ancient organelles thought to have emerged from the phagocytosis of alpha-protobacteria by eukaryotic cells long ago. Mitochondria contain their own closed double-stranded circular DNA (mtDNA) that is distinct from nuclear DNA (nDNA) [17]. Through evolutionary processes, the mtDNA lost the majority of its genes through genomic transfer and incorporation of cytoplasmic mtDNA into the nuclear genome. mtDNA is 16,569-base-pair long, void of introns, and has only a few noncoding nucleotides [18, 19]. mtDNA contains a total of 37 genes of which it encodes 13 essential proteins (mRNAs) of the oxidative phosphorylation pathway, along with 22 tRNAs that are found between most mRNA regions, as well as 2 rRNAs known as the 16S and 12S regions [19]. The presence of these tRNAs and rRNAs enables mitochondria to synthesize proteins within the organelle independently of the ER. The remainder of the proteins found in mitochondria are encoded in the nucleus, translated in the cytoplasm, and subsequently transferred to the mitochondria [20].

Mitochondrial functions range from mass production of ATP by oxidative phosphorylation, mediation of calcium signaling and sequestration, and regulation of apoptosis as well as OS by producing the highest cellular concentration of reactive oxygen species (ROS) as a metabolic byproduct [21, 22]. As the “power house” of the cell and an essential mediator of cellular metabolic homeostasis, mitochondrial dysfunction is implicated in aging and numerous age-related diseases [22].

## 4. HN and Related MDPs within the Mitochondrial Genome

**Figure 1:**
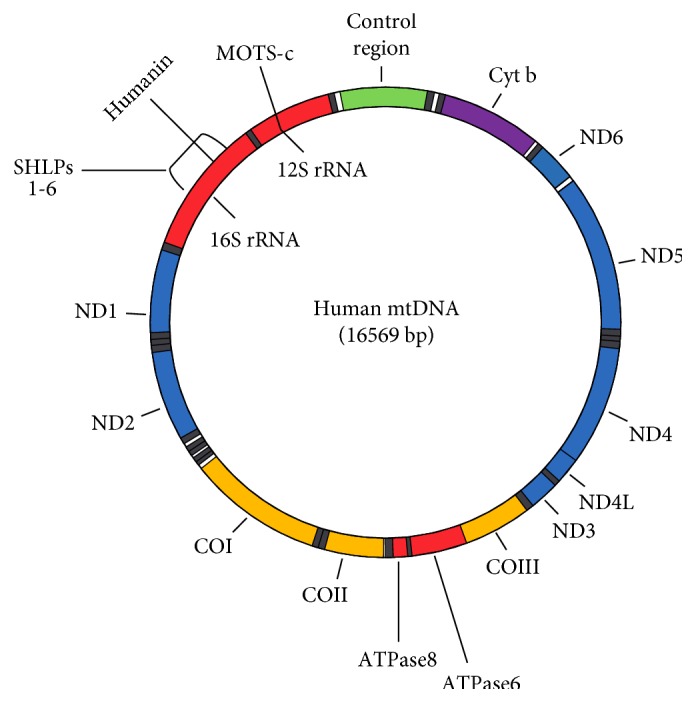
Human mtDNA genome showing location of humanin and SHLPs (16sRNA) and MOTS-c (12S rRNA). Regions for subunits of other proteins are also indicated in the figure. rRNA: ribosomal RNA; ND1 to ND6 and ND4: subunits of NADH dehydrogenase complex (complex 1); COI to COIII: subunits of cytochrome c oxidase (complex 1V); ATP6 and ATP8: subunits of ATP synthase; Cyt b: cytochrome b of CoQ-cytochrome c reductase (complex III).

High-resolution sequencing identified the existence of short open reading frames (sORFs) within the 16S and 12S rRNA regions of the mtDNA that were previously undetected [23, 24]. These sORFs lead to the discovery of MDPs that are encoded and transcribed within the mitochondria. To date, the known MDPs in order of their discovery include humanin (HN), mitochondrial open reading frame of the 12S rRNA-c (MOTS-c), and small humanin-like peptides (SHLPs) of which 6 variants have been identified [16, 25–28]. A pictorial representation of the mitochondrial genome depicting the MDPs in the 16S and 12S rRNA along with other encoded mitochondrial proteins is presented in Figure 1. The amino acid sequences of the MDPs are listed in Table 1.

HN is a highly conserved 75 base pair transcript encoded within the sORF of mitochondrial 16S rRNA [25, 29]. Interestingly, mitochondrial translational machinery yield a distinct number of peptide amino acids compared to cytoplasmic translation of the same gene. Therefore, depending on cytoplasmic versus mitochondrial locale, translation of HN yields either a 24- or 21-amino acid peptide, respectively [30, 31]. Both peptides have been shown to have biological activity [27]. Thirteen humanin-like ORFs were found within the nuclear genome: out of which, 10 were found to be expressed in tissue samples [32]. Additionally, two of these peptides were synthesized and their antiapoptotic properties were established [32].

## 5. Protective Properties of Humanin in Nonocular Tissues

HN was the first MDP discovered in 2001 by cloning a cDNA library to the 16S rRNA region to screen for molecules conferring neural apoptotic resistance against a mutated amyloid precursor protein (APP) from the unaffected portion of brain tissue of an Alzheimer disease (AD) patient [33, 34]. The peptide was also shown to confer neuroprotection against an array of familial Alzheimer's disease (FAD) genes including presenilin 1, presenilin 2, and mutated APP [25, 33]. HN was independently cloned by two additional groups when screening for various binding factors. Ikonen et al. [26] cloned HN as a binding partner of insulin-like growth factor binding protein-3 (IGFBP-3), while Guo et al. [27] discovered that HN binds to Bax and is a potent cytoprotective agent suppressing apoptosis. By binding to Bax, HN prevents its translocation and suppresses the downstream release of cytochrome c from the mitochondria thereby preventing apoptosis [27]. Additionally, HN binds to and deactivates the proapoptotic BH3 proteins tBid and BimEL [35, 36].

In glial cell lines, HN binding to IGFBP-3 blocks IGFBP-3-mediated apoptosis. However, in neurons, the cytoprotective capabilities of HN and IGF-1 synergize against A*β*-induced apoptosis [26, 30]. HN binding to IGFBP-3 affects the bioactivity of IGF-1 by increasing clearance without hindering IGFBP-3/IGF-1 binding and reduces circulating levels of IGFBP-3 and IGF-1 [37]. Both IGF-1 and HN inhibit apoptosis, enhance insulin sensitivity, suppress ischemia/reperfusion injury, lower inflammation, and degrade atherosclerotic plaques [37]. Conversely, HN and IGF-1 have opposite effects on longevity and tumorigenesis and both show a decline in expression with aging [37–39].

An age-related decline of HN could play a role in the pathogenesis of age-related diseases including AD and type 2 diabetes mellitus (T2DM) [39]. HN also inhibits pancreatic *β*-cell apoptosis, improves glucose tolerance, and lowers lymphocyte infiltrates in the nonobese diabetic (NOD) mouse model [40]. HN expression increases in response to stressful stimuli as evident from patients with mitochondrial encephalomyopathy with lactic acidosis and stroke-like episodes (MELAS) and localizes to the mitochondria to presumably increase ATP synthesis and improve cell survival [41].

In addition to FAD genes, HN confers neuroprotection against a variety of other degenerative diseases including spinocerebellar ataxia and Huntington's disease-related polyglutamine toxicity, disorders in which mitochondrial dysfunction has been implicated [30]. As a cytoprotective factor, HN also responded to A*β* and OS in triple transgenic mice by ameliorating cognitive impairment [30, 42]. Atherosclerotic plaques contain oxidized LDL aggregates similar to drusen, and these generate OS insults to the endothelial vasculature and HN suppressed the size of these plaques in ApoE-deficient mice on a high-fat diet [43]. Rat ischemia/reperfusion models also showed attenuated insults from cobalt chloride- (CoCl_2_-) mediated hypoxia and serum starvation-induced apoptosis by increasing mitochondrial respiration [30, 44]. Undifferentiated rat pheochromocytoma (PC12) cells were rescued from apoptosis under serum starvation conditions by HN and the potent HN analog, HNG [45]. In mouse cerebral artery stroke models, HNG reduced infarct volume by half when administered centrally and had similar effects with systemic injections as well [46].

## 6. Localization of HN and Its Putative Receptors in RPE Cells

HN is expressed in the cytoplasmic compartments in nonpolarized RPE cells where it is mainly localized in mitochondria [47]. In polarized RPE monolayer cultures which mimic the native and physiological RPE monolayer [48], HN did not exhibit polarized localization and was found in both the apical and basal compartments [47]. The three reported receptors, namely, ciliary neurotrophic factor receptor (CNTFR*α*), the cytokine receptor (WSX1), and the transmembrane glycoprotein gp130 (gp130) which are essential for the extracellular action of HN, were expressed in RPE cells [47]. All three receptors were expressed in both nonpolarized and polarized hRPE cells. CNTFR*α* and gp130 showed polarized localization, predominately localized to the apical domain, while WSX1 showed apical as well as basal localization. The binding of HN to heterotrimeric HN receptor (htHNR) results in oligomerization of the receptor subunits and subsequent activation of JAK2 and STAT3 [49–52]. Hashimoto et al. [49] showed that HN induces STAT3 phosphorylation, which was essential for its neuroprotective effects. Glycoprotein 130 is a common element of receptors that belong to the interleukin-6 (IL-6) receptor family and could trigger intracellular signal cascade responsible for (JAK)/STAT and ERK1/2 pathway in neuronal cells [52]. Ciliary neurotrophic factor receptor (CNTFR) is a known IL-6 family cytokine [49]. WSX-1 was found while testing gp130-coupling proteins that co-overexpressed with human gp130 [50]. *In vitro* pulldown analysis indicated that HN binds to CNTFR or WSX-1 [49]. HN treatment induced the dimerization between CNTFR and WSX-1 as well as the dimerization between WSX-1 and gp130. Thus, HN initially induces the dimerization between WSX-1 and CNTFR and then induces the hetero-trimerization of CNTFR/WSX-1/gp130 [49]. In addition, overexpression of CNTFR and/or WSX-1 results in enhanced HN binding to neuronal cells, whereas siRNA-mediated knockdown of both or either component reduces binding. Our study in RPE cells showed that HN activates phosphorylation of STAT3, and incubation with STAT3 inhibitor diminished the protective effect of HN significantly but not completely [47]. Therefore, it has to be reasoned that the receptor-mediated effects of HN peptide only partially contributed to the prevention of cell death. More recent work demonstrates that HN acts through the GP130/IL6ST receptor complex to activate AKT, ERK1/2, and STAT3 signaling pathways [53].

## 7. Endogenous Expression of HN and Its Functions

There are published reports that HN is expressed endogenously by several cells and tissues in the body such as cardiomyocytes, RPE cells, brain, colon, testis, heart, kidney, skeletal muscle, and liver [47, 54–57]. Further, endogenous HN has been reported to be secreted from cells [25, 29, 47] to the plasma [58–61] and transported to targeted tissues which express HN receptors. Overexpression of HN-protected cells from oxidant insult induced cell death [25, 27, 29]. Further, synthetic HN peptide mimicked the neuroprotection offered by HN-ORF cDNA at concentrations as low as that of secreted HN peptide in the culture medium [25, 29]. In addition, knockdown of endogenous HN by gene silencing correlated with increased sensitivity to oxidative stress- (OS-) induced cell death [27]. Polarization of RPE cells increased endogenous HN levels three-fold over nonpolarized RPE cells [47]. This increase was shown to be correlated with oxidative stress- (OS-) induced cell survival. These studies attest to the importance of HN in cell survival mechanisms.

## 8. Effects of Oxidative Stress in RPE

The retina is the most vascularized tissue in the human body by mass and is unique in that two independent circulatory networks—the central retinal artery and the choroidal vessels supply it to maintain its high metabolic demand [62]. As such, RPE cells are highly susceptible to oxidative stress by producing ROS as metabolic byproducts predominantly by robust mitochondrial oxygen consumption during cellular respiration and as a consequence of electron leakage from the respiratory chain enzymes [12]. The vulnerability of RPE cells is also as a result of the large oxygen gradient from the choroid, across the RPE to the outer retina [12]. ROS is also generated by the visual cycle as peroxidation products of photoreceptor polyunsaturated fatty acids and NADPH oxidase-mediated reactions during RPE phagocytosis and recycling of shed POS [12, 63].

Reactive oxygen species are essential physiological signaling molecules modulating gene expression, apoptosis, and proliferation, but are also toxic oxidizers of biomolecules and are linked to many pathologies such as Parkinson disease, AD, atherosclerosis, cancer, diabetes, and age-related diseases [64, 65]. In this regard, the cellular redox status represents a paradox in which an overabundance of oxidizers with insufficient reducing equivalents will accumulate ROS to concentrations that are maladaptive for cell survival by oxidizing and therefore perturbing the structure and function of lipids, proteins, and nucleic acids [21]. Consequently, retinal cells, as with all cells of the body, express enzymatic and nonenzymatic antioxidants and various modalities to repair and or replace oxidized material to circumvent dysfunction and ultimately apoptosis. RPE cells are particularly efficient at maintaining redox homeostasis thanks to, in part, DNA polymerases with sophisticated base excision repair (BER) exonuclease activity and an overabundance of enzymatic and nonenzymatic antioxidants such as reduced GSH and macular pigments that scavenge both photogenerated and nonphotic ROS [12, 66].

As alluded to earlier, the association between AMD and OS comes from biochemical evidence that the toxic, lipid-rich, granules deposited between the RPE and BM are mostly composed of oxidized proteins and lipids. A direct molecular connection between oxidative damage and AMD was established by the finding that carboxyethylpyrrole is elevated in BM and drusen from AMD patients [67]. The multipotent functions of HN and most of the cellular studies have been performed on neuronal cells and AD-related models. Although similarities in the pathogenesis of AD and AMD have been described [68], very little is known on the role of HN in AMD.

## 9. Exogenous HN Improves Mitochondrial Energetics in Oxidatively Stressed RPE

Due to its high metabolic activity, RPE cells harbor a large number of mitochondria which represent one of the major source of endogenous ROS. Mitochondria are highly susceptible to oxidative damage, and mitochondrial DNA repair in the RPE appears to be relatively very slow [69]. Since RPE cells are postmitotic, damaged mitochondria are not removed as quickly [70] leading to increased ROS production, which may further damage mitochondria [71]. Dysregulated mitochondria result in significantly low energy production and apoptosis, considered one of the initiating factors of AMD [72, 73]. How the MDPs influence the mitochondrial processes was hitherto unknown. Thus, Sreekumar et al. [47] tested HN's potential role in preserving mitochondrial bioenergetics and ROS inhibition in RPE cells. Oxidative stress augmented mitochondrial superoxide production, and HN cotreatment prominently inhibited ROS formation. It is of interest that in cardiac myoblasts stressed with H_2_O_2_, it was reported that HN analog (HNG) preserved mitochondrial membrane potential and mitochondrial structural integrity and inhibited mitochondrial swelling [74]. These authors further showed that pretreatment with a HN analog exerted cardio protective effects against myocardial ischemia and reperfusion injury in a mouse model [55].

Since it is known that OS affects mitochondria by decreased ATP production, how HN influences mitochondrial bioenergetics needed to be addressed. RPE cells were cotreated with HN (10 and 20 *μ*g) and 150 *μ*M tBH for 24 h. OS induced by tBH significantly decreased mitochondrial respiration, reserve capacity, and ATP production while HN cotreatment dose dependently increased all these parameters thereby restoring mitochondrial functions [47]. Further, it was shown that enhanced bioenergetics is due to increased mitochondrial biogenesis by HN as evidenced by transmission electron microscopy studies and mtDNA copy number [47].

One major consequence of OS is the initiation of cellular senescence. Premature senescence has been suggested as a potentially important pathophysiological mediator of RPE cell atrophy in GA [75, 76]. HN delayed OS-induced premature senescence in RPE cells as evidenced by the regulation of markers of senescence. HN treatment significantly reduced senescence-associated *β*-Gal-positive cells, *Apo J* transcripts, and p16^INK4a^ expression [47].

## 10. Effects of ER Stress in RPE and Protection by Exogenous HN

The endoplasmic reticulum (ER) is a large convoluted organelle containing within it the synthetic machinery necessary for producing proteins that it then posttranslationally modifies, folds, and secretes to the Golgi apparatus for further processing [77]. Additional functions include drug detoxification, carbohydrate metabolism, lipid biosynthesis, and regulation of calcium homeostasis as the ER is the major storage depot of intracellular calcium that is essential for calcium-dependent protein folding by ER chaperones [78].

Protein maturation is dependent on the capacity of the ER to fold peptides into their proper tertiary structures, and this requires that the folding within the ER lumen be conducted in an oxidizing environment to facilitate the formation of disulfide bonds [79]. As such, a redox status favoring an oxidizing luminal microenvironment is fundamental for this process. Perturbation of this microenvironment by various insults including viral infections, metabolic disturbances, and OS by excessive ROS results in calcium efflux from the ER and toxic unfolded/misfolded protein aggregates that increase the ER burden and cause ER stress [80].

To maintain proteostasis and cell function, the ER activates an adaptive quality control measure known as the unfolded protein response (UPR). The UPR is initiated by three independent transmembrane stress transducers: (1) inositol-requiring kinase-1 (IRE1), (2) RNA-activated protein kinase-like ER kinase (PERK), and (3) activating transcription factor-6 (ATF6) [81]. The collective activation of these signaling pathways causes suppression of mRNA translation to prevent further production of unfolded/misfolded proteins, refolding of misfolded proteins by upregulating ER chaperones, and induction of the ER-associated degradation (ERAD) system to eliminate toxic protein aggregates [82]. In this regard, the acute phase of the UPR promotes cell survival when ER stress is salvageable; however, sustained stress will induce caspase activation, mitochondrial dysfunction, and apoptosis to remove pathological cells and spare healthy ones [82–84].

Although the exact stress sensing mechanism of the UPR is yet to be understood, glucose-regulated protein-78 (GRP78), sometimes referred to as immunoglobulin-binding protein (BiP), is a stress-sensitive ER resident chaperone thought to regulate its activation [85]. Normally, GRP78 remains bound to the transmembrane stress transducers within the ER lumen keeping them in an inactive state [85]. The presence of stressful stimuli induces GRP78 to dissociate from the transducers resulting in their dimerization, autophosphorylation, and activation of their respective UPR pathways [86].

If prolonged ER stress exceeds the UPR's adaptive limitations for attenuating ER burden, the IRE1, PERK, and ATF6 prosurvival signals initiate both caspase-dependent and independent programmed cell death (PCD) pathways resulting in mitochondrial dysfunction and apoptosis [81, 83, 84]. Particularly in the retina, IRE1/TNF receptor-associated factor-2 (TRAF2)/apoptosis signal-regulating kinase-1 (ASK1)/c-Jun amino-terminal kinase (JNK) and PERK/eIF2*α*/ATF4/CHOP can elicit several AMD-related pathways via the induction of VEGF, CHOP, caspase 4, and NF-*κ*B [87].

Only recently, the effect of HN has been studied in the context of ER stress [11]. In human primary RPE cells exposed to multiple ER stressors (tunicamycin, brefeldin A, and thapsigargin), HN pretreatment offered a dose-dependent protection from cell death. The study found that HN treatment downregulated CHOP expression and decreased activated caspase 3 and caspase 4. Further, while TM treatment elevated mitochondrial ROS production and decreased mitochondrial glutathione (GSH), HN cotreatment inhibited ROS formation and restored GSH synthesis under the experimental conditions. While clearly GSH was involved in cellular protection, other antioxidants such as catalase, Trx1, Grx1, and Grx2 and SOD II did not show appreciable change with HN. The protective action of HN was not restricted to RPE cells as the authors showed that HN also protected U-251 glioma cells exposed to TM [11]. A potential role of HN in calcium metabolism in which HN in the ER could regulate intracellular calcium flux has been reported [57].

## 11. Endoplasmic Reticulum-Mitochondrial Crosstalk

ER stress and OS are participants in an array of physiological and pathophysiological conditions. The interaction between ER and mitochondria is evolving as a crucial factor in the regulation of the dynamic changes in motility, structure, and shape of these organelles. Studies on ER-mitochondria interactions in RPE and their modulation are scarce. In this context, the work of Matsunaga et al. [11] is relevant to further our understanding of ER stress-related mechanisms in the RPE and retina and potential roles of mitochondrial peptides in cellular protection. The study revealed that multiple ER stressors caused cell death by increasing mitochondrial ROS and activating downstream cell death pathways. Furthermore, ER and mitochondria interact both physiologically and functionally at sites called mitochondrial-associated membranes (MAMs) [88]. The contact sites between the ER and mitochondria have been measured to be 10–30 nm wide [89, 90]. MAMs facilitate interorganelle communication between the ER and mitochondria and are critical for lipid synthesis and transport, mitochondrial functions, the maintenance of calcium homeostasis, and apoptosis [91]. Many ER and mitochondria-associated proteins such as chaperones, protein kinases, and proteins regulating mitochondrial dynamics and morphology have been identified in MAMs, suggesting the major involvement of MAMs in all physiological processes [92]. Studies showed that ER Ca^2+^ channels, including the IP3Rs and the mitochondrial voltage-dependent anion channel, are rich in MAMs, which could facilitate Ca^2+^ flow between ER and mitochondria [92]. There is evidence to show that the ER-mitochondria contact sites are involved in autophagosome formation and that many proteins in the MAM compartments are necessary for autophagic vesicle formation [93–95]. In addition, ER-mitochondria contact sites are relevant to mitochondrial biogenesis based on the findings that mitochondrial fission occurs at areas of ER-mitochondria contacts [90, 96]. Further, mitofusin 2, which regulates mitochondrial fusion, is also proposed as a tethering protein that connects ER with mitochondria [97].

**Figure 2:**
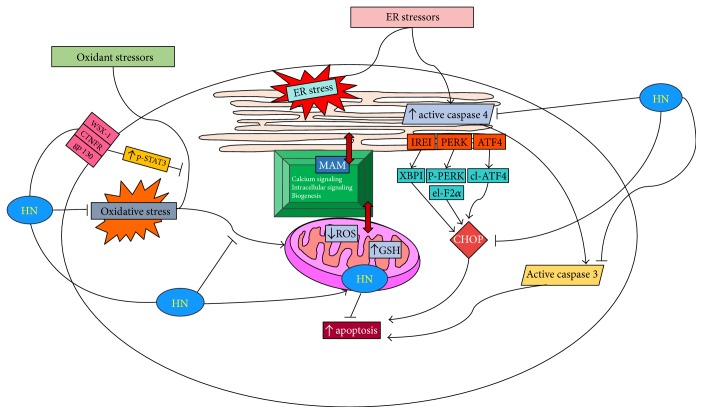
HN inhibits OS and ER stress in RPE by direct and indirect mechanisms. ER and mitochondria are linked through MAMs (mitochondria-associated membranes) which perform a variety of functions; some of which are listed in the figure. ER stress leads to CHOP induction via multiple signaling mechanisms. ER stress also activates procaspase 4 to active caspase 4 which in turn upregulates active caspase 3 leading to increased apoptosis. Exogenous HN's action on the suppression of ER stress-induced apoptosis via inhibition of caspase 4, CHOP, and caspase 3 are also shown in the figure. Exogenous HN is taken up by RPE and gains entry into mitochondria when cotreated with oxidant stressor. HN downregulates cellular OS and decreases mitochondrial ROS and augments mitochondrial GSH. The receptor-mediated pathway of HN preventing oxidant-induced apoptosis via activation of phosphorylated STAT3 is also shown.

Based on our findings, we present a composite scheme that depicts the ER-mitochondrial crosstalk in cellular stress and the protective role played by HN in RPE cell death by ER and oxidant stressors by direct and indirect mechanisms (Figure 2()). Both OS and ER stress cause RPE apoptosis by increased generation of ROS, downregulation of mitochondrial GSH, and activation of caspase 3 [11, 47]. Increased ER stress from OS via the crosstalk by MAMs and ER-specific events of caspase 4 and CHOP activation are also shown (Figure 2()). Uptake of exogenous HN by RPE mitochondria and the effect of exogenous HN cotreatment with oxidants or ER stressors on the inhibition of apoptotic cell death is illustrated. HN cotreatment inhibits ROS production and upregulates GSH in mitochondria. HN also inhibits caspase 4 and CHOP preventing RPE apoptosis. The known receptors of HN, namely, WSX-1, CNTFR, and gp130 and the inhibition of apoptosis by activation of p-STAT3 are also shown in the figure.

## 12. Future Directions

It would be of great interest to explore proteins and peptides including MDPs that affect ER stress-induced mitochondrial dysfunction and the related pathways participating in the process. On the other hand, the subcellular localization of HN is still under active investigation. We have provided evidence by confocal microscopy that fluorescein-labeled HN was rapidly taken by RPE and colocalized with mitochondria. However, it is not known whether HN is localized in ER compartments and this warrants additional studies. Further, the role of ER stressors on mitochondrial respiration and biogenesis in RPE and the nature of the salutary effects of HN will be an important issue to address. The role of ER stress on pro- and antiapoptotic factors in RPE cells such as Bax and Bcl-2 and the potential effect of HN on these factors remain to be studied. MAMs regulate several calcium-dependent cellular processes, and the movement of calcium between the ER and mitochondria is essential for the execution of both apoptotic [98] and autophagy pathways [99]. As stated earlier, HN was involved in calcium regulation [57], and detailed studies are needed to explore HNs' role in calcium regulation and autophagy under ER stress and OS. Additionally, the potential role of HN in regulating proteins present in MAMs would be of utmost interest to study given the recent studies suggesting the possible role of MAM protein role in mitochondrial dynamics [92]. ER stress and OS form a vicious cycle in human pathologies including AMD, and it would be of interest to determine the role of inflammatory molecules such as NLRP3 in AMD and how HN modulates these effects. Further, the recent discovery of additional MDPs, namely, SHLPs and MOTS-c, provides opportunity for exploration of these new MDPs in the therapy of AMD and related retinal degenerative disorders.

## 13. Therapeutic Potential of HN and Other MDPs in AMD

Since its discovery, HN has been validated to offer beneficial effects in many disease models, most of which are age-related. The advantage of HN as a therapeutic agent is that it antagonizes against a wide array of insults despite the complex nature of cytotoxic mechanisms. HN being a 24-amino acid peptide has rapid tissue clearance resulting in a very short half-life. To improve the half-life and to permit localized administration, we have suggested that making thermally responsive elastin-like polypeptides (ELP) [100] recombinantly conjugated with HN could be of value. Short peptides are typically proteolytically unstable and are cleared rapidly from circulation, and the ELP depot protects them from premature proteolysis and increases in vivo bioavailability [101]. Further, ELPs are useful and safe vehicles for both systemic and local drug delivery. Future studies will focus on evaluating the pharmacokinetics of ELP-MDPs for long term use.

## 14. Conclusions

Many recent studies indicate that ER stress and oxidative stress are highly interconnected biological processes which regulate a wide array of signaling pathways in the cell. Although it is known that both stress processes are closely associated, the mechanisms linking ER stress to OS are not fully explored. A greater understanding of the role of HN and MDPs on their mechanisms of action in the retina under pathophysiological conditions and development of optimal modes of their delivery will be of benefit in combating AMD and other related diseases.

## Figures and Tables

**Table 1:** Nomenclature and amino acid sequences of known MDPs.

Name	Sequence	Year discovered	References
HN	MAPRGFSCLLLLTSEIDLPVKRRA	2001, 2003	[25–27]
MOTS-c	MRWQEMGYIFYPRKLR	2015	[28]
SHLP1	MCHWAGGASNTGDARGDVFGKQAG	2016	[16]
SHLP2	MGVKFFTLSTRFFPSVQRAVPLWTNS	2016	[16]
SHLP3	MLGYNFSSFPCGTISIAPGFNFYRLYFIWVNGLAKVVW	2016	[16]
SHLP4	MLEVMFLVNRRGKICRVPFTFFNLSL	2016	[16]
SHLP5	MYCSEVGFCSEVAPTEIFNAGLVV	2016	[16]
SHLP6	MLDQDIPMVQPLLKVRLFND	2016	[16]

## Abbreviations

AD: Alzheimer disease
AMD: Age-related macular degeneration
APP: Amyloid precursor protein
ATP: Adenosine triphosphate
BER: Base excision repair
BM: Bruch membrane
CHOP: C/EBP homologous protein
CoCl_2_: Cobalt chloride
CNTFR: Ciliary neurotrophic factor receptor
CNV: Choroidal neovascularization
ER: Endoplasmic reticulum
ERAD: ER-associated degradation
ECM: Extracellular matrix
ERK1/2: Extracellular signal-regulated kinase 1/2
FAD: Familial Alzheimer's disease
GWAS: Genome-wide association studies
GA: Geographic atrophy
Gp130: Glycoprotein 130
HN: Humanin
htHNR: Heterotrimeric HN receptor
IGFBP-3: Insulin-like growth factor binding protein-3
MAMs: Mitochondrial-associated membranes
MDP: Mitochondrial-derived peptide
MOTS-c: Mitochondrial open reading frame of the 12S rRNA-c
mtDNA: Mitochondrial DNA
NOD: Nonobese diabetic
nDNA: Nuclear DNA
NUMT: Nuclear mitochondrial DNA transfer
OS: Oxidative stress
PC12: Pheochromocytoma cells
PCD: Programmed cell death
PI3K: Phosphoinositide-3-kinase
ROS: Reactive oxygen species
RPE: Retinal pigment epithelium
SHLPs: Small humanin-like peptides
sORF: Short open reading frame
STAT3: Signal transducer and activator or transcriptor 3
tBH: tert-Butyl hydroperoxide
T2DM: Type 2 diabetes mellitus
UPR: Unfolded protein response
VEGF: Vascular endothelial growth factor.
